# Ultrafast inactivation of SARS-CoV-2 with 266 nm lasers

**DOI:** 10.1038/s41598-022-23423-2

**Published:** 2022-11-04

**Authors:** Kexiong Sun, Gang Niu, Yanfang Zhang, Juan Yang, Danna Zhang, Han Wu, Xinyu Shao, Xiuquan Ma

**Affiliations:** 1 The State Key Laboratory of Digital Manufacturing Equipment and Technology, School of Mechanical Science and Engineering, Huazhong University of Science and Technology, Wuhan, Hubei China; 2 GZ Photonics Technology Co., Ltd., Dongguan, Guangdong China; 3 State Key Laboratory of Virology and National Virus Resource Centre, Wuhan Institute of Virology, Chinese Academy of Sciences, Wuhan, Hubei China; 4 Optics Valley Laboratory, Wuhan, Hubei China

**Keywords:** Microbiology, Optics and photonics

## Abstract

Disinfection eliminates pathogenic microorganisms and ensures a biosafe environment for human beings. The rapid spread of COVID-19 is challenging traditional disinfection methods in terms of reducing harmful side effects and conducting faster processes. Spraying large-scale chemical disinfectants is harmful to individuals and the environment, while UV lamp and light-emitting diode (LED) disinfection still requires a long exposure time due to the low irradiance and highly divergent beam characteristics. Given that a laser maintains a high irradiance over a long distance, we studied the effectiveness of lasers as a new disinfection method, and the results show the capability for ultrafast inactivation of SARS-CoV-2 virus with a 266 nm laser. This work confirms UV lasers as a good candidate for disinfection.

## Introduction

The novel coronavirus SARS-CoV-2 is responsible for a pandemic involving a serious respiratory disease that has spread worldwide. As of Mar 5th 2022, there have been over 440 million confirmed COVID-19 cases and more than 5.9 million reported deaths^1^. Even though vaccines and medications have been widely and successfully developed for COVID-19, the pandemic still spreads like wildfire. People have realized that for a pandemic disease such as COVID-19, disruption of the transmission chain still remains the most effective solution^2^.

The main transmission route of SARS-CoV-2 is by aerosols^3^ or by contact^4^. SARS-CoV-2 particles have been reported to be detectable for up to 3 h in aerosols^5^. Thus, close-distance aerosol transmission between humans and heating, ventilation, and air-conditioning (HVAC) system air exchange aerosol transmission between rooms are dangerous in terms of virus spreading^6–10^. Some research groups and institutions believe that aerosol transmission is being recognized as the dominant route for the COVID-19 pandemic, which has been shown in research studies^3^ and World Health Organization (WHO) statements^11^.

Traditional chemical methods are not applicable in such aerosol scenarios since the chemicals are either toxic or flammable in air. For example, medical alcohol is good for disinfection, but massive spraying of alcohol can cause fire and explosion. As another example, chemicals such as hydrogen peroxide, ozone and chlorine-based bleach are also good for disinfection, but they are toxic to the human body and should not be used for massive spraying around people. Therefore, spraying of chemicals to disinfect the air when people are around is not recommended under any circumstances^12^.

Especially for HVAC systems, no known methods are fast enough to disinfect aerosols in high-speed air flow conditions. Taking a central air-conditioner system as an example, the air flow speed is typically 20–30 m/s^13^. Thus, to disinfect the air flow between rooms in a central air-conditioning system, the disinfection should be finished in < 1 s for a 20 m long airduct, < 0.1 s for a 2 m long airduct, or < 0.01 s for a 0.2 m long airduct. Therefore, a safe (without toxic or flammable chemicals) and fast (fast enough to disinfect flowing air) disinfection method is needed.

Radiation-based inactivation methods are safe compared to toxic or flammable chemicals and are more convenient for inactivating SARS-CoV-2 and related coronaviruses according to former studies. Among all radiation-based inactivation methods, ultraviolet germicidal irradiation (UVGI) is the most extensively tested and widely used method for inactivating SARS-CoV-2^14^. UV light-emitting diodes (LEDs) and UV lamps are the most common candidates employed for UV light sources^15^. However, they both have some drawbacks. The LEDs have very low power efficiency; thus, they usually generate much heat and have quite a short practical lifetime. UV LEDs and lamps can only achieve > 99% virus inactivation after at least a few seconds to the best of our knowledge according to various publications^2,16–19^. This is because the light from regular incoherent light sources such as LEDs and lamps are very divergent when radiating, which leads to very low optical irradiance. Light from coherent light sources such as lasers can propagate without diverging, which leads to higher optical irradiance than regular incoherent light sources. This suggests that a UV laser with wavelength around 260 ~ 270 nm can be a good candidate for safe and fast virus disinfection. In this manuscript, we conduct a SARS-CoV-2 inactivation experiment with homemade 2 W 266 nm lasers, the results of which confirm the ultrafast performance of highly effective SARS-CoV-2 inactivation with 266 nm lasers.

## Calculation and comparison among lasers, LEDs and lamps

The disinfection performance follows an exponential decay law for the single-pass inactivation efficiency^20^:

1 $$\eta =1-{e}^{-k\cdot D},$$

where *η* is the inactivation efficiency, *k* is the UV rate constant (cm^2^/mJ) dependent on the virus species and wavelength, and *D* is the UV exposure dose (mJ/cm^2^). Therefore, the higher the UV exposure dose is, the higher the disinfection rate. The UV exposure dose *D* is the product of UV optical irradiance *I* (mW/cm^2^) and exposure time *t* (s):

2 $$D=I\cdot t,$$

where the UV optical irradiance is the optical power per unit area. Thus, the optical irradiance is proportional to the optical power divided by the optical beam area. This implies that if the optical beam area is more concentrated in a smaller area when the optical power remains the same, then the irradiance can dramatically increase, which directs our attention to lasers.

**Figure 1:**
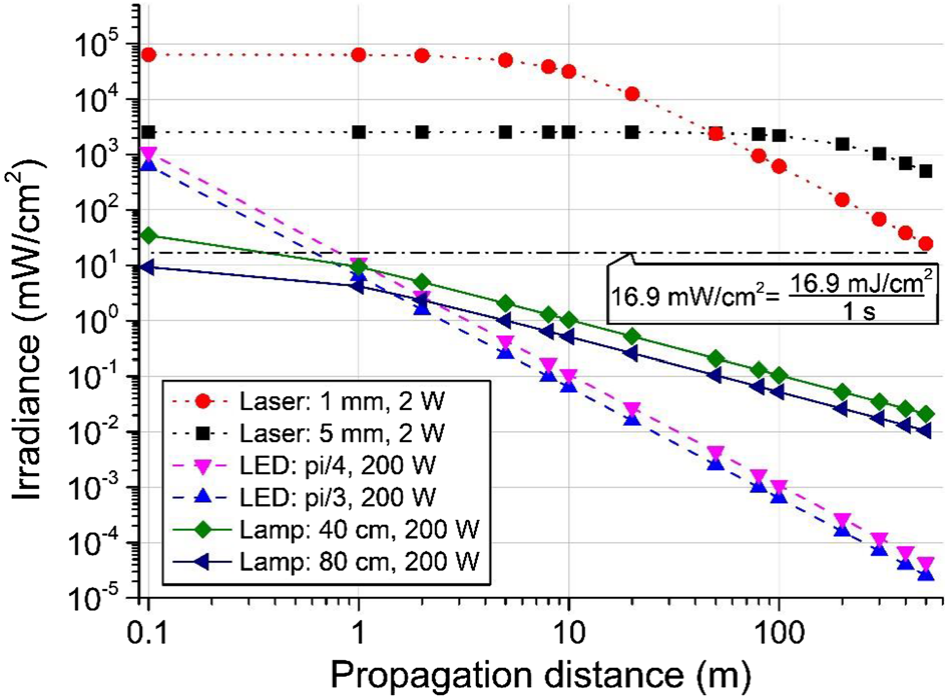
Irradiance with respect to propagation distance for (1) lasers at a power of 2 W with beam waist r = 1 mm and 5 mm, (2) LEDs at a power of 200 W with apex angle θ_half_ = 45° and 60°, and (3) lamps at a power of 200 W with length L = 40 cm and 80 cm. The black dotted-dashed horizontal line corresponds to the critical dose of 16.9 mJ/cm^2^ for SARS-CoV-2 inactivation when the exposure time is 1 s^21^.

Figure 1 illustrates the typical irradiance with respect to propagation distance for lasers, LEDs and lamps. Note that the black dotted-dashed horizontal line in Fig. 1 is the critical irradiance value of 16.9 mW/cm^2^ for the critical effective dose^21^ (there are multiple such values in the literatures and the 16.9 mJ/cm^2^ is picked as an example) at a 1 s exposure time for SARS-CoV-2 complete inactivation.

Unlike LEDs and lamps, lasers provide coherent light that propagates with a Gaussian wave front in the radial direction and a Gaussian profile in the axial direction. For example, for a high beam quality 266 nm laser, beam waist radii of 1 mm and 5 mm correspond to a Rayleigh range of approximately 10 m and 250 m, respectively, and the irradiance and propagation of such laser beams are shown in Fig. 1. The Rayleigh range is a measure of the distance within which a laser Gaussian beam almost maintains the same beam radius. Therefore, a laser beam with a waist radius of 5 mm can propagate a few hundred metres with no beam expansion, which is very different from the light sources people commonly use in everyday life, such as LED light sources and lamps.

In contrast to laser propagation, incoherent light, such as that from LEDs and lamps, constantly expands while propagating, so the irradiance is inversely proportional to the square of the propagation distance. For example, LED light sources commonly radiate light in a cone shape, which is commonly characterized by the cone apex angle, and the typical values are θ_half_ = 45° and 60°. Usually, one LED chip can emit 2 mW-10 mW, so a 200 W LED light source is composed of many single LED chips. Since we are considering the irradiance while the light propagates away from the LED source, the LED array can be simplified as a uniform distribution point source with a constant cone apex angle. The irradiance and propagation of a 200 W LED light source with θ_half_ = 45° and 60° are shown in Fig. 1. Very intuitively, the lamp radiates light in all directions, which can be seen as radiating almost over a 4π solid angle. However, for the purpose of obtaining a more accurate illustration of the lamp irradiance along the propagation direction, we apply the Keitz model to a long cylinder lamp bulb at 200 W power with length L = 40 cm and 80 cm^22^. The calculation results are also shown in Fig. 1.

Closely examining the black square dashed line in Fig. 1 representing the 266 nm laser beam with a 5 mm waist beam radius, the irradiance of this laser beam is more than 100 times higher than 16.9 mW/cm^2^ for a propagation range of more than 200 m. Therefore, in this 200-m propagation range, a less than 0.01 s exposure time can reach the critical effective dose of 16.9 mJ/cm^2^. This suggests that ultrafast inactivation of SARS-CoV-2 can be achieved with 266 nm lasers over a long span.

## Materials and methods

SARS-CoV-2/Wuhan/WIV04/2019 (SARS-CoV-2 WIV04) and SARS-CoV-2/630–1 (SARS-CoV-2 delta) were included in our experiments to verify the effectiveness of laser inactivation. Sindbis virus (SINV) with a similar molecular structure (also an enveloped RNA virus) but a different host cell was used to confirm that the surrounding environment may affect the viral sensitivity to laser irradiation. Pseudorabies virus (PRV) with DNA as the genetic material was used for comparison to RNA viruses given that the mechanisms’ difference between DNA and RNA damaged by UV, i.e. one of the mechanisms of UV irradiation disinfection for DNA is T-T dimer formation, while for RNA, it is U-U dimer formation. Human enterovirus 71 (EV71) and porcine parvovirus (PPV) with nonenveloped RNA/DNA were used to test any interactions between the viral envelope and UV photons that may affect the viral sensitivity.

### Cell lines and culture

The cell lines used in the experiments included Vero E6, BHK, PK15, ST and RD cells from the National Virus Resource Center of the Wuhan Institute of Virology, Chinese Academy of Sciences, which were cultured in minimum essential medium (MEM, Gibco™, Cat No: 42360032) supplemented with 10% foetal bovine serum (FBS) (Gibco, 10099–141) and 100 U/mL penicillin and streptomycin each (Gibco, 15140–122) at 37 °C in a 5% CO_2_ incubator. The viruses and cells used in our experiments are shown in Table 1.

**Table 1:** Viruses and cells used in the study.

Virus		Cells
SARS-CoV-2/Wuhan/WIV04/2019 (SARS-CoV-2 WIV04)	RNA enveloped	Vero E6 cells
SARS-CoV-2/630–1 (SARS-CoV-2 delta)	RNA enveloped	Vero E6 cells
Sindbis (SINV)	RNA enveloped	BHK cells
Pseudorabies (PRV)	DNA enveloped	PK15 cells
Human enterovirus 71 (EV71)	RNA nonenveloped	RD cells
Porcine parvovirus (PPV)	DNA nonenveloped	ST cells

### Virus proliferation

SARS-CoV-2 WIV04 and SARS-CoV-2 delta were propagated in Vero E6 cells. Vero E6 cells were seeded in a T-75 cell culture flask overnight, and the viruses were inoculated into the culture at a multiplicity of infection (MOI) = 0.1 when the cells were 80% confluent. The infected cell culture flask was placed in a 37℃ incubator for virus adsorption for 1 h, and the fresh medium of MEM + 2% FBS was replaced. Supernatants of infected cells were collected in a 15 mL centrifuge tube two days later. Cell fragments were discarded after centrifugation at 3000 r/min at 4 °C for 10 min, and viruses were obtained. The obtained viruses were separated and frozen in a refrigerator at − 80 °C for later use^23^.

Furthermore, SINV was propagated in BHK cells at 0.01 MOI, EV71 was propagated in RD cells at 0.01 MOI, PRV was propagated in PK15 cells at 0.1 MOI, and PPV was propagated in ST cells at 0.1 MOI, and they were harvested at 24 h, 24 h, 48 h and 72 h, respectively. The media they suspended was still MEM + 2% FBS. They were harvested in the same manner as SARS-CoV-2. All experiments involving SARS-CoV-2 experiments were performed in a biosafety 3 laboratory (P3), and SINV, PRV, EV71 and PPV experiments were performed in a biosafety 2 laboratory (P2). All viruses were obtained from the National Virus Resource Center of the Wuhan Institute of Virology, Chinese Academy of Sciences.

### Virus titration

Vero E6, BHK, RD, ST, and PK15 cells were seeded in 6-well plates overnight. The cell inoculation density was 10^4^ per well. A strain of the viruses stored in a refrigerator at − 80 °C was taken. After the virus thawed, MEM + 2% FBS was used to perform tenfold gradient dilution, with a total of 9 dilutions from 10^−1^ to 10^−9^, and 6–8 repetitions were made for each dilution. Three days after inoculation, the cytopathic effect (CPE) was scored, and the Reed-Muench formula was used to calculate the 50% tissue culture infectious dose (TCID_50_)^24^. The titre of the virus inoculum started at ~ 6 Lg TCID_50_/0.1 ml^25^. The lower limit of the titre was 1 Lg TCID_50_/0.1 ml (complete inactivated).

### Laser

**Figure 2:**
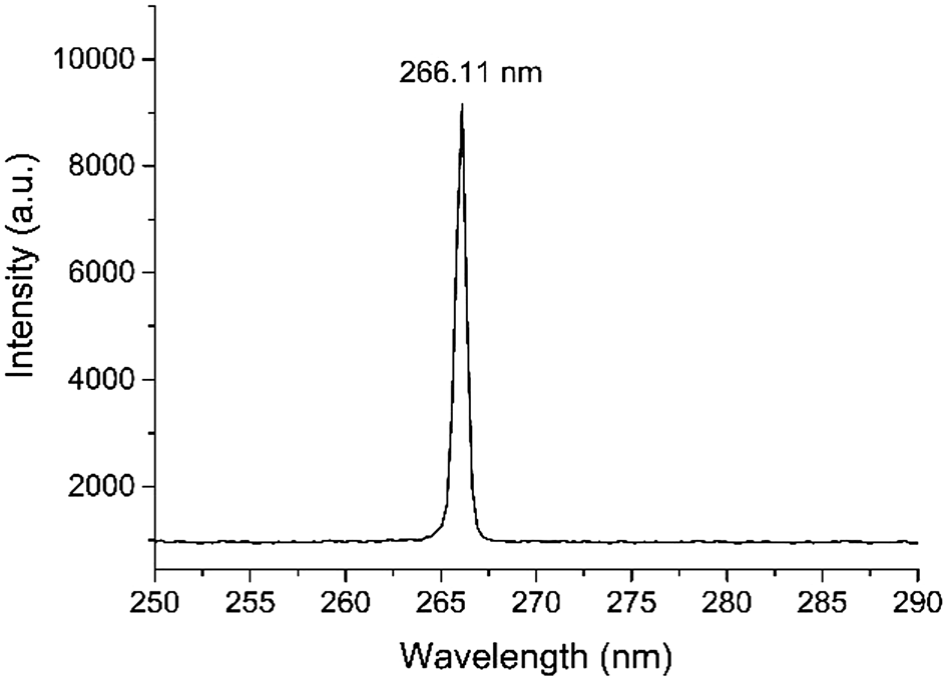
Spectra of the laser.

An in-house-built pulsed laser with a 10-ps pulse width, a 200-kHz repetition rate and an adjustable power output was used. The diameter of the beam is ~ 5.16 mm at the facet of the laser head and the beam divergence is ~ 18mrad. The wavelength tested was 266 nm, which is close to the RNA absorption maximum at approximately 260 nm^15^. The spectra of the laser is shown in Fig. 2 recorded by HR4000CG-UV-NIR (Ocean optics).

### Inactivation of SARS-CoV-2 by UV irradiation

A droplet of 0.1 mL of virus inoculum was placed in a defined well of a 6-well plate. Then, the laser head was positioned ~ 10 cm above the virus-containing well. The spot size of the laser was 0.785 cm^2^ or 0.601 cm^2^ (fully covering the surface area of the virus inoculum droplet in the well). After exposure for the designated time (controlled using a laser shutter, GCI-7102 M Daheng Optics Ltd.), each virus inoculum was subjected to virus titration. The number of replicates performed in our experiments is three.

### Data analysis

UV rate constant *k* is fitted from *η* = 1*−*e^*−kD*^, where inactivation efficiency *η* is the percentage of titre decreases and *D* is UV exposure dose. The k values are developed using the first 2 points for SARS-CoV-2 WIV04 and first 3 points for the other viruses. This is due to the titre for the third dose from small to large in SARS-CoV-2 WIV04 reaches the lower limit (1 Lg TCID50/0.1 ml), while in the other viruses this appears for the 4th dose. Once *k* is obtained, the dose required for the indicated inactivation efficiency, e.g., 90%, 99%, 99.9% and 99.99%, can also be calculated using *η* = 1*−*e^*−kD*^. The time required for the indicated inactivation efficiency is calculated using *D* = *I*∙*t*.

The absorptions and reflections of the MEM media and the 6-well plate are included as Supplementary Material 1. The percentage of energy absorbed in MEM media is 39.69% in our experimental analysis. The absorbed laser dose *D*_*absorbed*_ could be calculated as *D*_*absorbed*_ = 39.69% *D*_*irradiated*_ where *D*_*irradiated*_ is the irradiated laser dose. UV rate constant *k*_*absorbed*_ can be easily calculated as *k*_*absorbed*_ = *k*_*irradiated*_/39.69%. For simplicity, we did not do the conversion and only use *D*_*irradiated*_ and *k*_*irradiated*_ as *D* and *k* in the following analysis.

## Results and discussion

**Figure 3:**
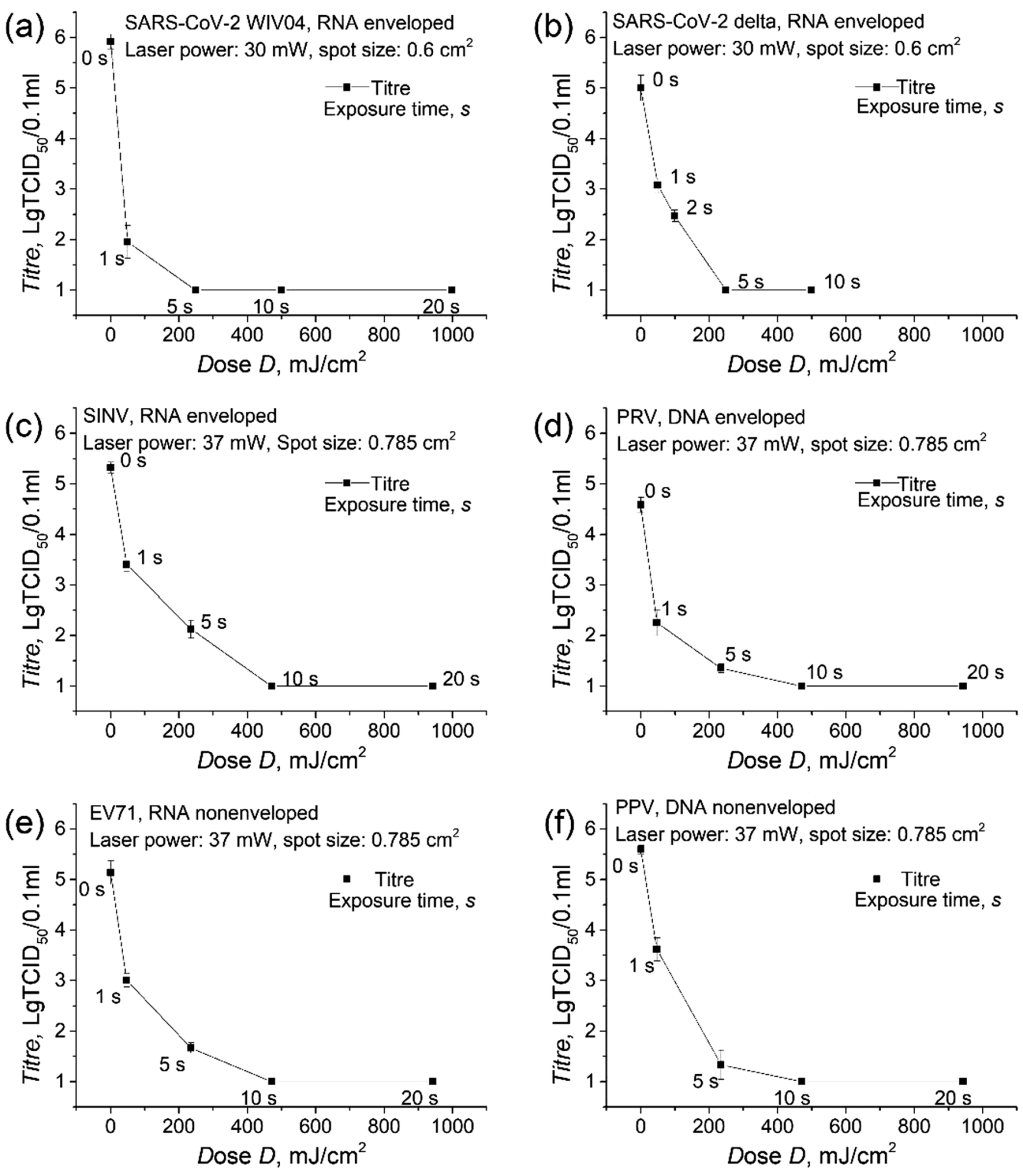
Virus inoculum titre with respect to the irradiation dose. The virus inoculum samples were irradiated by a 266 nm laser at a series of exposure times, and then, titre analysis was performed by virus titration. (**a**) SARS-CoV-2 WIV04 virus, (**b**) SARS-CoV-2 delta virus, (**c**) SINV, (**d**) PRV, (**e**) EV71, and (**f**) PPV.

To verify the effectiveness and calculate the species-dependent UV rate constant, 266 nm laser inactivation of the viruses at a series of exposure times was tested, as shown in Fig. 3. The raw data and calculation of the inactivation experiments was included as Supplementary Material 2. First, SARS-CoV-2 WIV04 and SARS-CoV-2 delta virus inactivation are reported in Fig. 3a,b. The pulsed 266 nm laser was able to achieve ~ 99% inactivation (corresponding to a decrease of ~ 2 Lg TCID_50_/0.1 ml) at 1 s and complete inactivation (down to 1 Lg TCID_50_/0.1 ml) after 5 s. This is our first step to confirm the validity of the laser approach. Second, SINV was used to check the effectiveness for non-SARS-CoV-2 viruses, as shown in Fig. 3c. SINV is an ~ 70-nm single-stranded enveloped RNA virus with a genome of 11.7 kb, which is comparable to SARS-CoV-2 (100 ~ 150 nm single-strand enveloped RNA virus, 27 ~ 32 kb genome). The pulsed 266 nm laser was able to achieve ~ 99% inactivation at 1 s and complete inactivation after 10 s. Finally, the type of virus in the verification experiments was extended to DNA enveloped (PRV), RNA nonenveloped (EV71), and DNA nonenveloped (PPV). The results showed the achievement of approximately 99% inactivation for all four of these viruses after 1 s of irradiation and complete inactivation after 10 s, as shown in Fig. 3d–f. Together, these results show that all six tested viruses are highly susceptible to pulsed 266 nm UV laser irradiation.

To imply the applicability of this method in the real world, the UV rate constants, doses and exposure times for achieving various reduction levels were calculated, as shown in Table 2. The UV rate constant is the core parameter in disinfection system design. Microbial susceptibility to UV light is evaluated using the UV rate constant *k*, which correlates the inactivation efficiency with the UV dose. Each kind of virus corresponds to a particular *k*. High rate constant values imply a lower dose required for a certain inactivation efficiency and vice versa. The doses required for achieving different reduction levels were comparable to those obtained in various publications using UV lamps and LEDs^16,21,26–28^. This indicates the susceptibility of SARS-CoV-2 and other viruses to the pulsed 266 nm laser. A pulsed 266 nm laser with high irradiance (usually 2 ~ 3 orders of magnitude higher than the irradiance of UV lamps and LEDs) may be a promising strategy for high-speed disinfection.

**Table 2:** Dose and exposure times for achieving various reduction levels.

Virus		SARS-CoV-2 WIV04	SARS-CoV-2 delta	SINV	PRV	EV71	PPV
*k* (cm^2^/mJ)		0.1759 ± 0.0093	0.0584 ± 0.0037	0.0281 ± 0.0023	0.0257 ± 0.0017	0.0289 ± 0.0023	0.0377 ± 0.0039
Dose (mJ/cm^2^) required for the indicated inactivation efficiency	90%	13.1176 ± 0.7026	39.5173 ± 2.5937	82.2628 ± 6.5332	89.8574 ± 6.3132	80.0555 ± 6.0773	61.4794 ± 6.1211
	99%	26.2352 ± 1.4053	79.0346 ± 5.1875	164.5256 ± 13.0665	179.7147 ± 12.6265	160.111 ± 12.1546	122.9588 ± 12.2422
	99.9%	39.3527 ± 2.1079	118.5519 ± 7.7812	246.7883 ± 19.5997	269.5721 ± 18.9397	240.1664 ± 18.2319	184.4382 ± 18.3634
	99.99%	52.4703 ± 2.8106	158.0692 ± 10.375	329.0511 ± 26.1329	359.4294 ± 25.2529	320.2219 ± 24.3092	245.9176 ± 24.4845
Time (s) required for the indicated inactivation efficiency	90%	0.2624 ± 0.0141	0.7903 ± 0.0519	1.7453 ± 0.1386	1.9064 ± 0.1339	1.6985 ± 0.1289	1.3044 ± 0.1299
	99%	0.5247 ± 0.0281	1.5807 ± 0.1037	3.4906 ± 0.2772	3.8129 ± 0.2679	3.3969 ± 0.2579	2.6087 ± 0.2597
	99.9%	0.7871 ± 0.0422	2.371 ± 0.1556	5.2359 ± 0.4158	5.7193 ± 0.4018	5.0954 ± 0.3868	3.9131 ± 0.3896
	99.99%	1.0494 ± 0.0562	3.1614 ± 0.2075	6.9812 ± 0.5544	7.6257 ± 0.5358	6.7939 ± 0.5157	5.2174 ± 0.5195

By one-way ANOVA with Tukey's post-test on k values, we found that there is statistically significant difference (P < 0.0001) among the six kinds of virus. Specially, the k values of SARS-CoV-2 WIV04 (Adj. P < 0.0001 for all comparisons) and SARS-CoV-2 delta (Adj. P < 0.00153 for all comparisons) is significantly higher than that in SINV, PRV, EV71 and PPV respectively. Particularly, the SARS-CoV-2 WIV04 exhibited significant higher k values than the SARS-CoV-2 delta (Adj. P < 0.0001). It is demonstrated that SARS-CoV-2 showed a better 266 nm laser inactivation sensitivity among the six kinds of virus, while SARS-CoV-2 WIV04 performed best. There were no significant differences (Adj. P > 0.06846 for all comparisons) on the k values between the SINV, PRV, EV71 and PPV group, indicating a close 266 nm laser inactivation sensitivity among them. By one-way ANOVA, there was no significant difference (P = 0.13951) between DNA and RNA virus. Also, by one-way ANOVA, there was no significant difference (P = 0.16562) between enveloped and non-enveloped virus.

## Conclusion

First, we proposed that a 266 nm laser can be a good candidate for safe and fast virus disinfection based on calculations and a comparison among lasers, LEDs and lamps. Next, we showed the ultrafast performance and high effectiveness of pulsed 266 nm laser inactivation of the SARS-CoV-2 virus through experiments. The pulsed 266 nm laser was able to achieve ~ 99% inactivation at a 1 s exposure time for SARS-CoV-2 WIV04 and SARS-CoV-2 delta virus. SINV (RNA enveloped), PRV (DNA enveloped), EV71 (RNA nonenveloped) and PPV (DNA nonenveloped) are also highly susceptible to pulsed 266 nm UV laser irradiation, indicating a universal effect of UV laser disinfection. The ultrafast inactivation of SARS-CoV-2 and other viruses is attributable to the high irradiance of the laser relative to lamps and LEDs. Finally, the UV rate constants, doses and exposure times for achieving various reduction levels were calculated and can be used as core parameters in future disinfection system design or other applications. This work indicates a promising prospect that 266 nm laser inactivation is fast enough to disinfect flowing air in a single pass. Further verification experiments, such as aerosol disinfection and chamber tests, should be conducted. The pulse duration and repetition rate may influence the inactivation efficiency and bring difference in the inactivation mechanism, which is worth investigate in the future.

To further develop lasers as a practical ultrafast disinfection method and apply them to real-world HVAC systems, the small area of a laser beam (result in small disinfecting areas or volumes) remains one of the primary limitations (the other main issue is cost). The solution should be expansion and shaping of laser beam. The price of this is the large decrease of irradiance (inversely proportional to the square of spot radius). To compensate the decrease in irradiance, the power of laser should be increased (which will further aggravate the cost issue). Therefore, generating laser active zone that meet the designed disinfection volume and dose at a reasonable cost is the key to success in developing laser to be a practical disinfection method. The necessary things to do are optimal design of beam shaping to achieve large active area, minimum power loss of optical components selection and reduction of laser cost per watt.

## Supplementary Information

Supplementary Information 1.

Supplementary Information 2.

## Data Availability

The datasets used and/or analysed during the current study are included in this published article and its supplementary information files. All data generated or analysed during this study are available from the corresponding author on reasonable request.
