# Supplementary material for: Ultrafast inactivation of SARS-CoV-2 with 266 nm lasers
Source: Sci Rep. 2022 Nov 4;12:18640. doi: 10.1038/s41598-022-23423-2 (PMC9636154; doi:10.1038/s41598-022-23423-2)
Supplement: Supplementary file 1 — Supplementary Information 1. [file 41598_2022_23423_MOESM1_ESM.docx]

Supplementary material 1

The absorptions and reflections of the MEM media and the 6-well plate

The transmittance (Figure S1) at normal incidence of MEM media and the reflectance (Figure S2) at normal incidence from air to MEM media is recorded using spectrophotometer (SolidSpec-3700 UV-2) which is available for liquid sample.


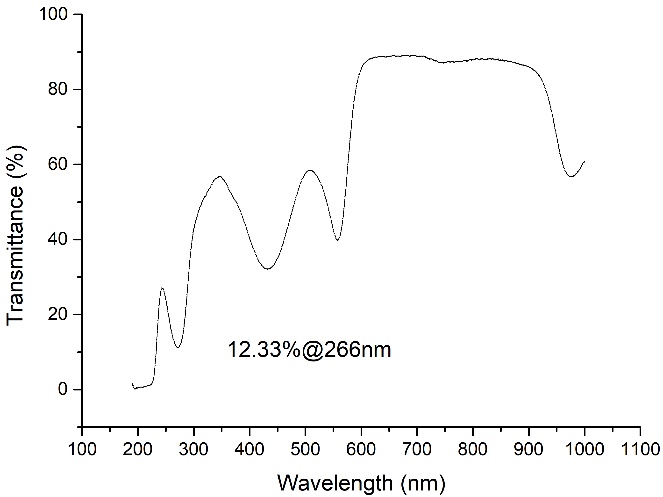


Figure S1 MEM media transmittance (1cm depth) at normal incidence

According to Beer-Lambert law *I_o_=I_i_exp(-αl)* where *I_o_* is the output beam, *I_i_* is the incident beam, *l* is the absorption length, *α*=-*Ln*(*I_o_/I_i_)/l* is the absorption coefficient. In Figure S1 (*I_o_/I_i_=12.33%@266nm)*, the absorption coefficient of MEM media transmittance at normal incidence is calculated as 0.208 mm^-1^.


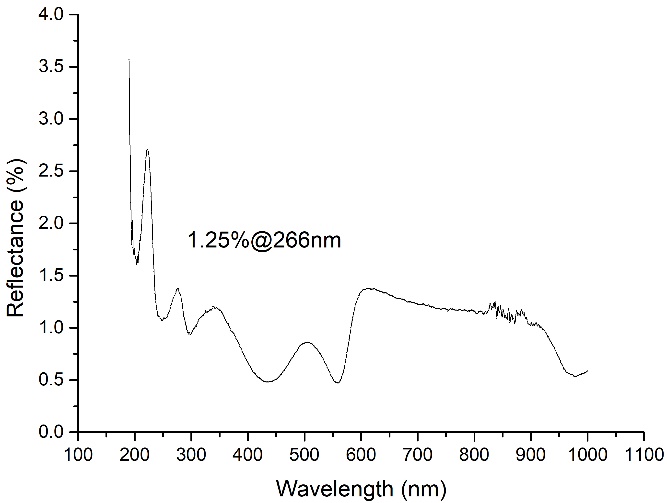


Figure S2 MEM media reflectance at normal incidence

The reflectance at 266nm normal incidence from air to MEM media is 1.25% as shown in Figure S2.


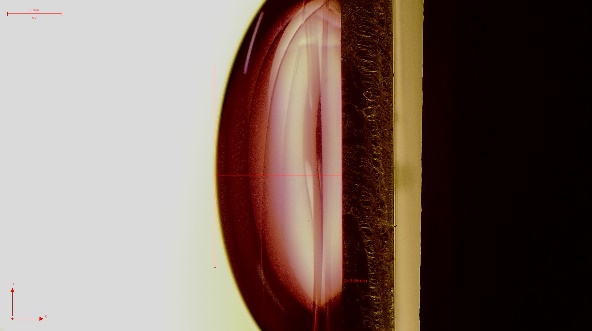


Figure S3 0.1mL MEM media droplet on the 6-well plate. The height of the droplet is 2.36mm.

The transmittance at 266nm normal incidence of MEM media droplet with height of 2.36mm (Figure S3) is 61.21%. The absorption at 266nm normal incidence of MEM media droplet with height of 2.36mm is 1-1.25%-61.21%=37.54%.

Table S1 reflectance @266nm of inner well surface of 6-well plate

|  | Well #1 | Well #2 | Well #3 | Well #4 | Well #5 | Well #6 | Mean |
| --- | --- | --- | --- | --- | --- | --- | --- |
| reflectance | 7.892% | 8.982% | 8.8696% | 9.0534% | 9.019% | 7.8074% | 8.6039% |

In Table S1, the reflectance from air to 6-well plate @266nm of inner well surface of 6-well plate is recorded using spectrophotometer (lambda 750S) which is available for solid sample.

The reflectance of normal incidence (regardless of the s or p polarization) is calculated as R=(n_2_-n_1_)^2^/(n_2_+n_1_)^2^ where n_2_ is the refractive index of transmitted media and n_1_ is the refractive index of incident media. The reflectance at 266nm normal incidence from air(refractive index n=1) to MEM media is 1.25% as shown in Figure S2. So, the refractive index of MEM media is calculated as 1.2517. The reflectance at 266nm normal incidence from air(refractive index n=1) to 6-well plate is 8.6039% as shown in Table S1. So, the refractive index of 6-well plate is calculated as 1.83. The reflectance at 266nm normal incidence from MEM media (refractive index n=1.2517) to 6-well plate (refractive index n=1.83) is calculated as 3.52%.

100%

1.25% Reflect

37.54% Absorption

**Air**

**MEM media**

**6-well plate**

(1-1.25%-37.54%) *3.52% Reflect

(1-1.25%-37.54%) *(1-3.52%) Transmit

Figure S4 The energy path from air to MEN media to 6-well plate.

Considering the 1^st^ order reflectance, the energy path is shown in Figure S4. The percentage of energy absorbed in MEM media is 39.69%. The absorbed laser dose *D_absorbed_* could be calculated as *D_absorbed_* =39.69%*D_irradiated_* where *D_irradiated_* is the irradiated laser dose. UV rate constant *k_absorbed_* can be easily calculated as *k_absorbed_* =*k_irradiated_*/39.69%. For simplicity, we did not do the conversion and only use *D_irradiated_* and *k_irradiated_* as *D* and *k*.

Note that this analysis does not consider the MEM media as a droplet. There will be non-normal incidence components on the curved surface of a droplet. Further accurate calculation will need to take this into account. Current analysis has helped to get the general idea about where the energy goes.
