# Supplementary material for: Ultrafast inactivation of SARS-CoV-2 with 266 nm lasers
Source: Sci Rep. 2022 Nov 4;12:18640. doi: 10.1038/s41598-022-23423-2 (PMC9636154; doi:10.1038/s41598-022-23423-2)
Supplement: Supplementary file 2 — Supplementary Information 2. [file 41598_2022_23423_MOESM2_ESM.docx]

Supplementary material 2

The raw data and calculation of the inactivation experiments

Table S2 The raw data and calculation. The k values were developed using the first 2 points for SARS-CoV-2 WIV04 and first 3 points for the other viruses.

| SARS-CoV-2 WIV04 | | | Test & Calculation Replicate | | |  |  |
| --- | --- | --- | --- | --- | --- | --- | --- |
|  |  |  | #1 | #2 | #3 | Mean | SD |
| Dose (mJ/cm^2^) | 0.00 | Titre (LgTCID_50_/0.1ml） | 6.00 | 5.75 | 6.00 | 5.9167 | 0.1443 |
|  | 49.92 |  | 2.40 | 1.75 | 2.16 | 2.1033 | 0.3287 |
|  | 249.58 |  | 1.00 | 1.00 | 1.00 | 1.0000 | 0.0000 |
|  | 499.17 |  | 1.00 | 1.00 | 1.00 | 1.0000 | 0.0000 |
|  | 998.34 |  | 1.00 | 1.00 | 1.00 | 1.0000 | 0.0000 |
| k (cm^2^/mJ) | | | 0.1660 | 0.1845 | 0.1771 | 0.1759 | 0.0093 |
| Dose required for the indicated inactivation efficiency (mJ/cm^2^) | | 90.00% | 13.8710 | 12.4801 | 13.0016 | 13.1176 | 0.7026 |
|  |  | 99.00% | 27.7420 | 24.9603 | 26.0032 | 26.2352 | 1.4053 |
|  |  | 99.90% | 41.6130 | 37.4404 | 39.0048 | 39.3527 | 2.1079 |
|  |  | 99.99% | 55.4840 | 49.9205 | 52.0064 | 52.4703 | 2.8106 |
| Time required for the indicated inactivation efficiency (s) | | 90.00% | 0.2774 | 0.2496 | 0.2600 | 0.2624 | 0.0141 |
|  |  | 99.00% | 0.5548 | 0.4992 | 0.5201 | 0.5247 | 0.0281 |
|  |  | 99.90% | 0.8323 | 0.7488 | 0.7801 | 0.7871 | 0.0422 |
|  |  | 99.99% | 1.1097 | 0.9984 | 1.0401 | 1.0494 | 0.0562 |
| SARS-CoV-2 delta | | | Test & Calculation Replicate | | |  |  |
|  |  |  | #1 | #2 | #3 | Mean | SD |
| Dose (mJ/cm^2^) | 0.00 | Titre (LgTCID_50_/0.1ml） | 4.75 | 5.00 | 5.25 | 5.0000 | 0.2500 |
|  | 49.92 |  | 3.25 | 3.00 | 3.00 | 3.0833 | 0.1443 |
|  | 99.83 |  | 2.40 | 2.40 | 2.60 | 2.4667 | 0.1155 |
|  | 249.58 |  | 1.00 | 1.00 | 1.00 | 1.0000 | 0.0000 |
|  | 499.17 |  | 1.00 | 1.00 | 1.00 | 1.0000 | 0.0000 |
| k (cm^2^/mJ) | | | 0.0542 | 0.0600 | 0.0611 | 0.0584 | 0.0037 |
| Dose required for the indicated inactivation efficiency (mJ/cm^2^) | | 90.00% | 42.4831 | 38.3956 | 37.6732 | 39.5173 | 2.5937 |
|  |  | 99.00% | 84.9662 | 76.7912 | 75.3464 | 79.0346 | 5.1875 |
|  |  | 99.90% | 127.4494 | 115.1868 | 113.0196 | 118.5519 | 7.7812 |
|  |  | 99.99% | 169.9325 | 153.5825 | 150.6927 | 158.0692 | 10.3750 |
| Time required for the indicated inactivation efficiency (s) | | 90.00% | 0.8497 | 0.7679 | 0.7535 | 0.7903 | 0.0519 |
|  |  | 99.00% | 1.6993 | 1.5358 | 1.5069 | 1.5807 | 0.1037 |
|  |  | 99.90% | 2.5490 | 2.3037 | 2.2604 | 2.3710 | 0.1556 |
|  |  | 99.99% | 3.3986 | 3.0716 | 3.0139 | 3.1614 | 0.2075 |
| SINV | | | Test & Calculation Replicate | | |  |  |
|  |  |  | #1 | #2 | #3 | Mean | SD |
| Dose (mJ/cm^2^) | 0.00 | Titre (LgTCID_50_/0.1ml） | 5.40 | 5.25 | 5.03 | 5.2267 | 0.1861 |
|  | 49.92 |  | 3.50 | 3.31 | 2.50 | 3.1033 | 0.5311 |
|  | 249.58 |  | 2.25 | 2.00 | 1.25 | 1.8333 | 0.5204 |
|  | 499.17 |  | 1.00 | 1.00 | 1.00 | 1.0000 | 0.0000 |
|  | 998.34 |  | 1.00 | 1.00 | 1.00 | 1.0000 | 0.0000 |
| k (cm^2^/mJ) | | | 0.0264 | 0.0273 | 0.0307 | 0.0281 | 0.0023 |
| Dose required for the indicated inactivation efficiency (mJ/cm^2^) | | 90.00% | 87.3846 | 84.4985 | 74.9052 | 82.2628 | 6.5332 |
|  |  | 99.00% | 174.7693 | 168.9971 | 149.8104 | 164.5256 | 13.0665 |
|  |  | 99.90% | 262.1539 | 253.4956 | 224.7155 | 246.7883 | 19.5997 |
|  |  | 99.99% | 349.5385 | 337.9941 | 299.6207 | 329.0511 | 26.1329 |
| Time required for the indicated inactivation efficiency (s) | | 90.00% | 1.8540 | 1.7927 | 1.5892 | 1.7453 | 0.1386 |
|  |  | 99.00% | 3.7079 | 3.5855 | 3.1784 | 3.4906 | 0.2772 |
|  |  | 99.90% | 5.5619 | 5.3782 | 4.7676 | 5.2359 | 0.4158 |
|  |  | 99.99% | 7.4159 | 7.1710 | 6.3568 | 6.9812 | 0.5544 |
| PRV | | | Test & Calculation Replicate | | |  |  |
|  |  |  | #1 | #2 | #3 | Mean | SD |
| Dose (mJ/cm^2^) | 0.00 | Titre (LgTCID_50_/0.1ml） | 4.50 | 4.75 | 4.50 | 4.5833 | 0.1443 |
|  | 49.92 |  | 2.50 | 2.25 | 2.00 | 2.2500 | 0.2500 |
|  | 249.58 |  | 1.25 | 1.40 | 1.40 | 1.3500 | 0.0866 |
|  | 499.17 |  | 1.00 | 1.00 | 1.00 | 1.0000 | 0.0000 |
|  | 998.34 |  | 1.00 | 1.00 | 1.00 | 1.0000 | 0.0000 |
| k (cm^2^/mJ) | | | 0.0270 | 0.0264 | 0.0237 | 0.0257 | 0.0017 |
| Dose required for the indicated inactivation efficiency (mJ/cm^2^) | | 90.00% | 85.1548 | 87.3846 | 97.0327 | 89.8574 | 6.3132 |
|  |  | 99.00% | 170.3095 | 174.7693 | 194.0653 | 179.7147 | 12.6265 |
|  |  | 99.90% | 255.4643 | 262.1539 | 291.0980 | 269.5721 | 18.9397 |
|  |  | 99.99% | 340.6191 | 349.5385 | 388.1307 | 359.4294 | 25.2529 |
| Time required for the indicated inactivation efficiency (s) | | 90.00% | 1.8067 | 1.8540 | 2.0587 | 1.9064 | 0.1339 |
|  |  | 99.00% | 3.6133 | 3.7079 | 4.1173 | 3.8129 | 0.2679 |
|  |  | 99.90% | 5.4200 | 5.5619 | 6.1760 | 5.7193 | 0.4018 |
|  |  | 99.99% | 7.2266 | 7.4159 | 8.2347 | 7.6257 | 0.5358 |
| EV71 | | | Test & Calculation Replicate | | |  |  |
|  |  |  | #1 | #2 | #3 | Mean | SD |
| Dose (mJ/cm^2^) | 0.00 | Titre (LgTCID_50_/0.1ml） | 5.40 | 5.00 | 5.00 | 5.1333 | 0.2309 |
|  | 49.92 |  | 2.88 | 3.15 | 3.00 | 3.0100 | 0.1353 |
|  | 249.58 |  | 1.56 | 1.69 | 1.75 | 1.6667 | 0.0971 |
|  | 499.17 |  | 1.00 | 1.00 | 1.00 | 1.0000 | 0.0000 |
|  | 998.34 |  | 1.00 | 1.00 | 1.00 | 1.0000 | 0.0000 |
| k (cm^2^/mJ) | | | 0.0314 | 0.0282 | 0.0270 | 0.0289 | 0.0023 |
| Dose required for the indicated inactivation efficiency (mJ/cm^2^) | | 90.00% | 73.3307 | 81.6809 | 85.1548 | 80.0555 | 6.0773 |
|  |  | 99.00% | 146.6615 | 163.3618 | 170.3095 | 160.1110 | 12.1546 |
|  |  | 99.90% | 219.9922 | 245.0428 | 255.4643 | 240.1664 | 18.2319 |
|  |  | 99.99% | 293.3229 | 326.7237 | 340.6191 | 320.2219 | 24.3092 |
| Time required for the indicated inactivation efficiency (s) | | 90.00% | 1.5558 | 1.7330 | 1.8067 | 1.6985 | 0.1289 |
|  |  | 99.00% | 3.1116 | 3.4659 | 3.6133 | 3.3969 | 0.2579 |
|  |  | 99.90% | 4.6674 | 5.1989 | 5.4200 | 5.0954 | 0.3868 |
|  |  | 99.99% | 6.2232 | 6.9318 | 7.2266 | 6.7939 | 0.5157 |
| PPV | | | Test & Calculation Replicate | | |  |  |
|  |  |  | #1 | #2 | #3 | Mean | SD |
| Dose (mJ/cm^2^) | 0.00 | Titre (LgTCID_50_/0.1ml） | 5.50 | 5.64 | 5.64 | 5.5933 | 0.0808 |
|  | 49.92 |  | 3.36 | 3.75 | 3.75 | 3.6200 | 0.2252 |
|  | 249.58 |  | 1.50 | 1.50 | 1.00 | 1.3333 | 0.2887 |
|  | 499.17 |  | 1.00 | 1.00 | 1.00 | 1.0000 | 0.0000 |
|  | 998.34 |  | 1.00 | 1.00 | 1.00 | 1.0000 | 0.0000 |
| k (cm^2^/mJ) | | | 0.0344 | 0.0367 | 0.0420 | 0.0377 | 0.0039 |
| Dose required for the indicated inactivation efficiency (mJ/cm^2^) | | 90.00% | 66.9162 | 62.6724 | 54.8496 | 61.4794 | 6.1211 |
|  |  | 99.00% | 133.8323 | 125.3449 | 109.6991 | 122.9588 | 12.2422 |
|  |  | 99.90% | 200.7485 | 188.0173 | 164.5487 | 184.4382 | 18.3634 |
|  |  | 99.99% | 267.6646 | 250.6897 | 219.3983 | 245.9176 | 24.4845 |
| Time required for the indicated inactivation efficiency (s) | | 90.00% | 1.4197 | 1.3297 | 1.1637 | 1.3044 | 0.1299 |
|  |  | 99.00% | 2.8394 | 2.6593 | 2.3274 | 2.6087 | 0.2597 |
|  |  | 99.90% | 4.2591 | 3.9890 | 3.4911 | 3.9131 | 0.3896 |
|  |  | 99.99% | 5.6788 | 5.3187 | 4.6548 | 5.2174 | 0.5195 |
